# Plasma miR-203a-3p as a Novel Predictor of Dementia in Patients with Parkinson’s Disease

**DOI:** 10.3390/ijms25063554

**Published:** 2024-03-21

**Authors:** Ya-Fang Hsu, Shau-Ping Lin, Yung-Tsai Chu, Yi-Tzang Tsai, Jing-Wen Huang, Frederick Kin Hing Phoa, Ruey-Meei Wu

**Affiliations:** 1 Graduate Institute of Brain and Mind Sciences, College of Medicine, National Taiwan University, Taipei 100225, Taiwan; r09454013@ntu.edu.tw; 2 Department of Neurology, National Taiwan University Hospital, College of Medicine, National Taiwan University, Taipei 100225, Taiwan; chuyungtsai@gmail.com; 3 Institute of Biotechnology, College of Bio-Resources and Agriculture, National Taiwan University, Taipei 106038, Taiwan; shaupinglin@ntu.edu.tw (S.-P.L.);; 4 Department of Neurology, National Taiwan University Hospital Jinshan Branch, New Taipei City 208204, Taiwan; 5 Centre for Parkinson and Movement Disorders, National Taiwan University Hospital, Taipei 100225, Taiwan; 6 Institute of Statistics, National Tsing Hua University, Hsinchu 300044, Taiwan; miffy8485@gmail.com; 7 Institute of Statistics, Academia Sinica, Taipei 115201, Taiwan; fredphoa@stat.sinica.edu.tw

**Keywords:** plasma biomarker, microRNA, Parkinson’s disease, cognitive decline, droplet digital PCR

## Abstract

The early detection of cognitive decline in Parkinson’s disease is important for providing drug therapy and non-pharmacological management. The circulating microRNAs present in plasma are promising biomarkers of PD with dementia (PDD) due to their critical roles in synaptic plasticity and the regulation of neurodegeneration-associated proteins. In this study, we aimed to identify plasma microRNAs that may differentiate PD with or without cognitive impairment. Global microRNA expression was obtained from a discovery set of 123 participants who were divided into four groups, namely normal controls (HC), PD with no dementia (PDND), PD with mild cognitive impairment (PD-MCI), and PDD, using next-generation sequencing. The BOLD selector was used for microRNA candidate selection. Six miRNAs, namely miR-203a-3p, miR-626, miR-662, miR-3182, miR-4274, and miR-4295, were clustered as potential candidates for use in identifying PDND from PD-MCI. Another independent cohort of 120 participants was further recruited in a validation step in order to detect candidate microRNAs via droplet digital PCR (ddPCR), which was used for its high sensitivity in detecting low miRNA concentrations. Our results show that the ratio of miR-203a-3p/miR-16-5p, in which miR-16-5p was used as a reference control miRNA, was significantly increased in PDD compared to that seen in PD-MCI and PDND individually, and was negatively correlated with the MoCA scores (r = −0.237, *p* = 0.024) in patients with PD. However, there was no significant difference in the ratio of miR-203a-3p/miR-16-5p between HC and PDND, PD-MCI, or PDD individually. The ROC curve of the logistic regression model, factoring in the variables of age, the ratio of miR-203a-3p/miR-16-5p, and the UPDRS III score, demonstrated an AUC of 0.883. Our findings suggest that the ratio of miR-203a-3p/miR-16-5p, used with age and motor score, could be a predictor of dementia among PD patients.

## 1. Introduction

Parkinson’s disease (PD) is the second most common neurodegenerative disorder in the elderly population [1]. The clinical features of PD not only include motor symptoms, such as bradykinesia, tremor, and postural rigidity, but also non-motor symptoms, including fatigue, apathy, and cognitive dysfunction [2]. It was reported that approximately 26.7% of PD patients are diagnosed with mild cognitive impairment (PD-MCI) [3], and around 20–60% of PD-MCI patients may convert into cases of PD with dementia (PDD) within five years of diagnosis [4]. The incidence rate of developing dementia is higher in patients with PD (PwP) than in non-PD controls [5]. Histologically, Lewy bodies, mainly consisting of the fibril alpha-synuclein (α-syn) present in the substantia nigra pars compacta, are well-known pathological hallmarks of PwP [2]. The pathological changes in α-syn, amyloid-β (Aβ) plaques, and tau neurofibrillary tangles (NFTs) found in PDD have been reported to contribute to the cognitive decline in PwP [6]. Motor dysfunction with cognitive dementia could increase the economic and psychological burden for caregivers [7]. PwP with dementia will gradually lose their basic living ability and may have shorter lifespans than individuals with PD without dementia [5]. This could become a serious issue for individuals and societies dealing with an aging population [8,9]. Thus, the identification of clinical and biological markers that can be used for the detection or prediction of the severity of cognitive decline in PwP is critical for referring the patients to clinical trials of PDD. Moreover, patients with PDD and PD-MCI may display different prognoses throughout the course of the disease and exhibit diverse responses to new drugs during clinical trials. Patients diagnosed with PDD would have the opportunity to rearrange their life for the benefit of their well-being and safety.

In clinical practice relating to the diagnosis of PDD, neuropsychological tests [10]; regular examinations, including blood testing; and brain imaging techniques, such as magnetic resonance imaging (MRI) [11], are commonly used. However, the entire procedure of cognitive examination is often time-consuming and requires the involvement of multiple medical personnel [10,11]. Recently, emerging molecular biomarkers of inherited genetic mutants, including *GBA* and *SNCA*, or toxic proteomic markers, such as abnormal aggregated α-syn, have attracted attention regarding their influence on PD pathology, and these may be utilized to identify PwP who are at risk of cognitive decline [12,13,14]. Compared with tissue biopsy or cerebrospinal fluid (CSF) collection methods, non-invasive methods for plasma collection have caused it to become one of the most commonly studied resources of human biomarkers [15].

MicroRNAs (miRNAs) are single-stranded non-coding RNAs with an average length of 22 nucleotides [16]. MiRNAs can mediate post-transcriptional expression via binding with target messenger RNAs (mRNAs) [17] and cease the transcription of the encoded gene. MiRNAs exist not only in the cytoplasm but also in extracellular areas, such as the CSF, blood, and other biofluids [17,18]. Circulating miRNAs consist of different fractions of miRNAs [19] and may be contained within the extracellular vesicles and be transported across the blood–brain barrier (BBB) [20].

A miRNA candidate exploration study may include both a discovery study phase and a validation study phase. To explore new miRNA biomarkers for use identifying PD patients with cognitive decline in our cohort, we first conducted a discovery phase in order to acquire the global microRNA expression in a set of participants divided into four groups, namely normal controls (HC), PD with no dementia (PDND), PD with mild cognitive impairment, and PDD, using next-generation sequencing (NGS) and selected the potential candidate microRNAs by means of data analysis. Then, we conducted a validation phase by using droplet digital PCR (ddPCR) for the measurement of the candidate microRNAs selected in the discovery phase of a new independent cohort of controls and PD patients, both with and without cognitive impairment, included at the same hospital. We aimed to identify novel plasma miRNA candidates as biomarkers for use in the diagnosis of PD with cognitive impairment.

## 2. Results

### 2.1. Using Small RNA-Seq to Explore the Potential miRNA Candidates That May Allow for the Differentiation of PD with or without Cognitive Impairment

#### 2.1.1. Analyzing miRNA Candidates via NGS Profiling in the Discovery Cohort

We first performed a discovery study phase by using NGS (small RNA-seq) to explore the potential miRNA candidates associated with cognitive decline. The demographic variables of the discovery cohort are summarized in Table 1. The definitions and annotations of the miRNA species were based on their alignment with the miRNA reference database miRBase. The miRNA raw read counts detected across samples were normalized using a weighted trimmed mean of the log_2_ expression ratios (trimmed mean of M values, TMM). The results consisted of approximately 2600 miRNAs and were filtered through a series of data processing steps and statistical analyses via the Biomedical Oriented Logistic Dantzig (BOLD) selector [21]. The BOLD selector was used for miRNA candidate selection when considering the supersaturated data, and we used it to analyze the thousands of miRNA profiles obtained from the relatively small sample. The transformation of nonlinear programming into the linear programming framework during the BOLD selector analytic scheme was supposed to increase the efficiency of the traditional statistical method. The binary response, classified as either the experimental group or the control group provided by the logistic regression formula, was used for ROC curve analysis in a 5-fold cross-validation test.

**Figure 1:**
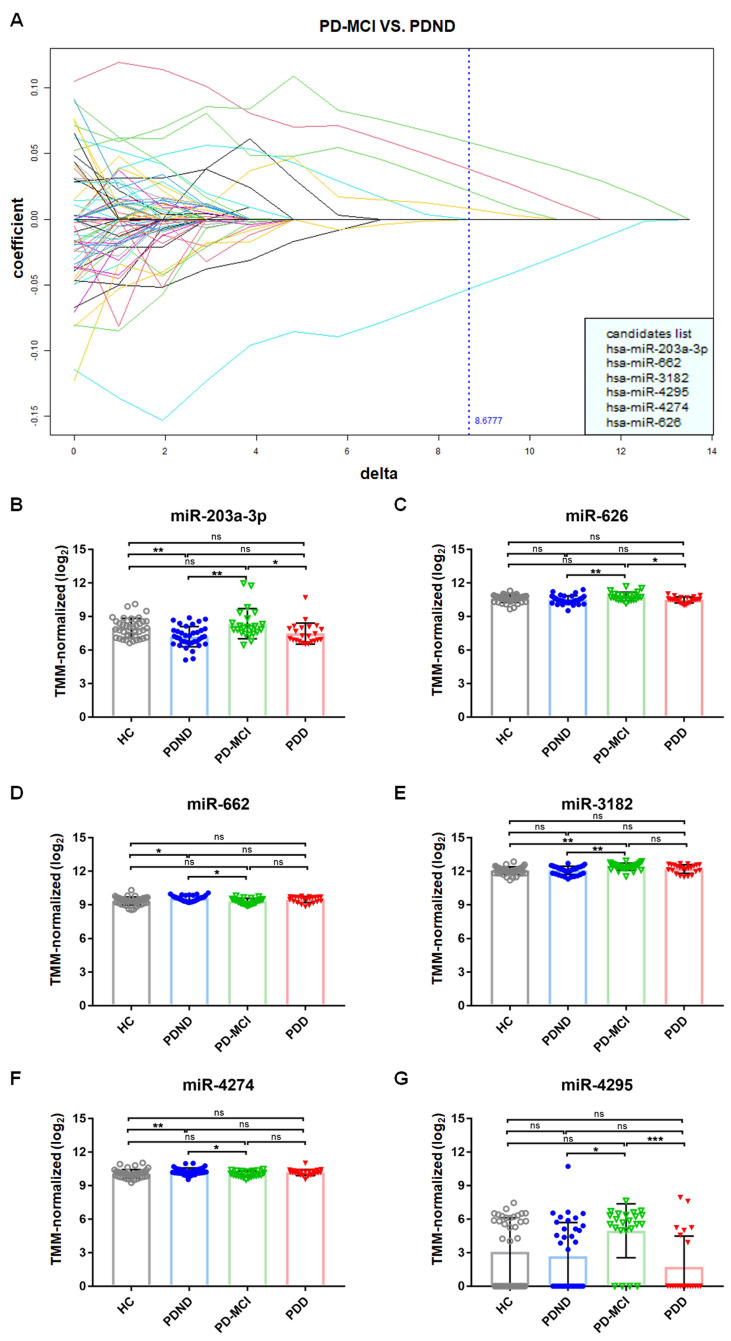
miRNA candidates selected via BOLD selector statistical analysis. HC, healthy control; PDND, Parkinson’s disease with no dementia; PD-MCI, Parkinson’s disease with mild cognitive impairment; PDD, Parkinson’s disease with dementia. (**A**) The *x*-axis is the value of the tuning parameter δ, which roughly translates to the increase in noise when considering the ability of biomarkers to differentiate the two disease conditions. The *y*-axis is its corresponding solution in linear programming, which in this case represents the coefficient of each biomarker candidate shown in different color of lines for differentiating PD-MCI (*n* = 23) from PDND (*n* = 37) under conditions of continuous change in the δ value. We argue that a candidate is important if its eventual shrink-to-zero position is equal to or larger than the selected δ (the blue dotted line). We rank the importance of candidates based on their shrink-to-zero orders. In particular, the last candidate to shrink to zero is implied to be the most important one, and the second to last takes second place, and so on. (**B**–**G**) We performed Kruskal–Wallis tests followed by Dunn’s multiple comparisons tests (post hoc test) for the comparison of the miRNA expression level between the different study groups in the discovery phase. HC (*n* = 40), PDND (*n* = 37), PD-MCI (*n* = 23), and PDD (*n* = 23). Data are shown as mean ± SD. The *y*-axis indicates the NGS reads of the miRNA; the *x*-axis indicates the diagnosis group of each sample. (**B**) The TMM-normalized NGS reads (log_2_) of miR-203a-3p. (**C**) The TMM-normalized NGS reads (log_2_) of miR-626. (**D**) The TMM-normalized NGS reads (log_2_) of miR-662. (**E**) The TMM-normalized NGS reads (log_2_) of miR-3182. (**F**) The TMM-normalized NGS reads (log_2_) of miR-4274. (**G**) The TMM-normalized NGS reads (log_2_) of miR-4295. * *p*-value < 0.05; ** *p*-value < 0.01; *** *p*-value < 0.01; ns, no significant difference.

Eventually, six miRNAs, namely miR-203a-3p, miR-626, miR-662, miR-3182, miR-4274, and miR-4295, were clustered as potential candidates for use identifying PDND from PD-MCI (Figure 1A). The heat map plot shows the six miRNA candidates (TMM-normalized log_2_ reads), which included all samples from the four study groups (HC, PDND, PD-MCI, and PDD) present in the discovery phase (Figure S1). Using Kruskal–Wallis tests, we found that the NGS reads of the individual miRNAs showed significant differences between the four study groups (Figure 1B–G). Four of the six miRNAs, including miR-203a-3p, miR-626, miR-3182 and miR-4295, were upregulated, while the other two miRNAs, miR-662 and miR-4274, were downregulated in PD-MCI compared to their presence in PDND. In the discovery cohort, we compared the expression level of miRNAs between PDND and PDD. However, the selected candidates from that comparison did not have high predicting power (AUC < 0.7). Hence, we did not identify any other candidates that could be used to distinguish between PDD and PDND. The fold change in miRNA reads was calculated as log_2_ × (the mean of the patient group with cognitive decline/the mean of the patient group without cognitive decline). When comparing PD-MCI with PDND, the fold change (log_2_FC) in the miRNA expression level was 0.218 for miR-203a-3p, 0.044 for miR-626, -0.038 for miR-662, 0.037 for miR-3182, −0.034 for miR-4274, and 0.884 for miR-4295 (Figure 1B–G). However, no significant differences were observed in the expression of the six miRNA candidates together between PDND and PDD. The above findings encouraged us to carry out a follow-up investigation of these plasma miRNAs in a new PwP cohort with a larger sample size, and we validated the differential power of the miRNA expression level in cases of PD with different cognitive statuses. The failure to differentiate PDD from PDND in the discovery cohort may have been due to the low sensitivity of cognitive assessments performed using the total score of the Mini-Mental Status Examination (MMSE) [22]. Hence, in the validation cohort, we evaluated the cognitive function of the PwP using the total score in the Montreal Cognitive Assessment (MoCA), which may provide better sensitivity in identifying early cognitive decline in PwP [23].

#### 2.1.2. Measurement of the Selected miRNA Candidates

We then aimed to validate the previous findings by recruiting another PwP cohort, which was used as the validation cohort. In our literature review, we found that miR-4274 has been reported in diseases associated with esophageal squamous cell carcinoma [24] and normal-pressure hydrocephalus [25], but not neurodegeneration. We therefore did not rank this microRNA as a high priority in the validation phase. Furthermore, approximately half of the samples in the discovery cohort failed to display miR-4295. As a result, we selected the other four miRNAs, namely miR-203a-3p, miR-626, miR-662, and miR-3182, in the validation phase. The four miRNA candidates were first quantified using locked nucleic acid (LNA)-based RT-PCR (Qiagen Sciences, Germantown, MD, USA), and melt curve analysis was performed for the purposes of validating the specificity of the RT-PCR assay. However, except for miR-203a-3p, the detection of the remaining three miRNA candidates via RT-PCR (Ct > 36) or non-overlapping dissociation curves failed. The failure in detecting miR-626, miR-662, and miR-3182 may have resulted from the different detection sensitivities between the NGS and RT-PCR platforms [17] and the low concentration of the total extracted plasma RNA, which was lower than the detection limit of the Bioanalyzer RNA Nano system. 

### 2.2. Validation of the miRNA Candidates in another PwP Cohort

#### 2.2.1. Motor Function Deterioration Was Associated with a Poor Cognitive Status

In Table 2, we summarize demographic variables such as the gender, age, MoCA score, education level, onset age, duration, Hoehn–Yahr stage, Unified Parkinson’s Disease Rating Scale (UPDRS) III, and the levodopa equivalent daily dose (LEDD) values of each study group. The MoCA scale was evaluated to assess the cognitive performance, such as the visuospatial or memory functions, of each participant. The Hoehn–Yahr stage is a system for describing the severity of symptoms in PD. The UPDRS III is the third part of the UPDRS scale used for the motor examination of patients. The LEDD value is calculated as a sum of anti-Parkinson drugs due to variability in medications and dose. The percentage of males in HC and PDND was 56.67%, while the percentage of males in PD-MCI and PDD was 53.33% and 46.67%, respectively. Those in PDD groups were older than those in HC (*p*-value < 0.0001) and PDND (*p*-value = 0.025) groups, while no significant difference in age was observed among HC, PDND, and PD-MCI groups. Although the duration of disease and the age of onset showed no significant differences among the PD patients, PDD had a significantly higher Hoehn–Yahr stage value than PDND (*p*-value < 0.0001) and PD-MCI (*p*-value = 0.0048). Correspondingly, PDD also had a higher UPDRS III score than PDND (*p*-value < 0.0001) and PD-MCI (*p*-value = 0.0002). This suggests that motor and cognitive functions may deteriorate more rapidly in PDD, given that the disease durations were similar across PDND, PD-MCI, and PDD. No significant difference was observed in the years spent in formal education among the HC, PDND, and PD-MCI groups. The PDD group had significantly fewer years of education than HC (*p*-value = 0.0256) and PDND (*p*-value = 0.0327) cohorts. Moreover, the value of LEDD was estimated according to the anti-Parkinson drug prescribed to the PD patients. The results show no significant difference in LEDD among PDND, PD-MCI, and PDD groups. Although anticholinergic drugs were excluded from the LEDD estimation, it was estimated that five patients of PDND, seven patients of PD-MCI, and one patient of PDD received a low-dose anticholinergic drug (2–4 mg/d) for the management of severe tremors.

#### 2.2.2. The Expression Level of Plasma miR-203a-3p/miR-16-5p Validated Using ddPCR

Because the total amount of circulating miRNA may differ from person to person and usually presents a very low concentration, and because quantification with high sensitivity and specificity was required, we used ddPCR to quantify the expression level of plasma miR-203a-3p in each fixed-volume sample [26]. Additionally, an endogenous reference miRNA was required for the normalization of the expression level of miR-203a-3p [27]. Endogenous miR-16-5p, which is abundant across intracellular and intercellular regions and has a relatively consistent presence in biofluids at different ages, has been used to normalize miRNA in studies of Parkinson’s disease and multiple-system atrophy [28,29,30]. Therefore, we combined the detection of our target miRNA and the endogenous reference miRNA into the ratio of miR-203a-3p/miR-16-5p in order to reduce individual biases that may not relate to PD pathologies. An exogenous synthetic oligonucleotide, UniSp6, was used as a reference for RNA extraction. The ratio of miR-16-5p/UniSp6 showed no significant difference among the study groups, suggesting the robust extraction efficiency of each sample (Figure S2). Examples of the ddPCR results and the expression level of miR-203a-3p among each study group are visualized in Figure S3.

**Figure 2:**
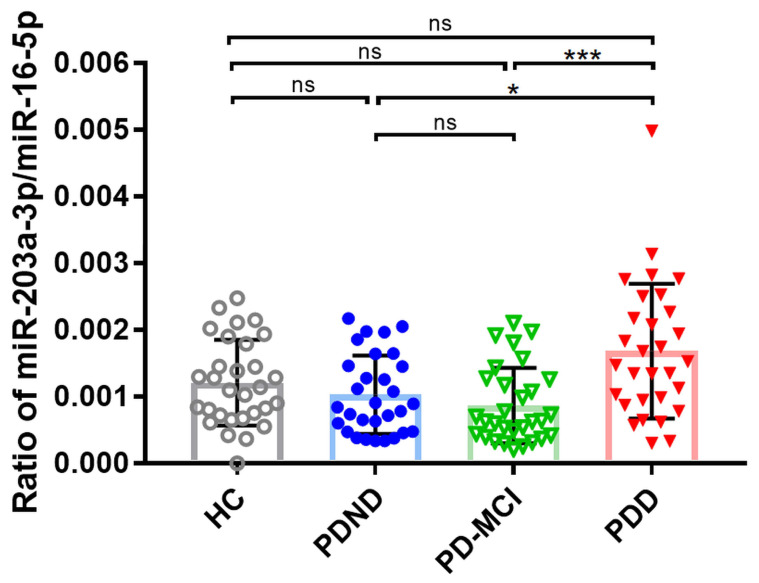
The ddPCR examination of miR-203a-3p/miR-16-5p. The ratio of miR-203a-3p/miR-16-5p of all samples in the validation cohort was analyzed using the Kruskal–Wallis test followed by Dunn’s multiple comparisons test (post hoc test) among the study groups. * *p*-value < 0.05; *** *p*-value < 0.001; ns, no significant difference. HC (*n* = 30), PDND (*n* = 30), PD-MCI (*n* = 30), and PDD (*n* = 30). Data are shown as the mean ± SD. HC, healthy control; PDND, Parkinson’s disease with no dementia; PD-MCI, Parkinson’s disease with mild cognitive impairment; PDD, Parkinson’s disease with dementia.

To elucidate whether the ratio of miR-203a-3p/miR-16-5p was different among the PD patients with and without cognitive decline, a non-parametric one-way ANOVA test, namely the Kruskal–Wallis test, was conducted (Figure 2). The results show that there were significant differences in the ratio of miR-203a-3p/miR-16-5p among the four groups (*p*-value = 0.0009). The mean ratio of miR-203-3p/miR-16-5p was 1.21 × 10^−3^ (SD, ±6.42 × 10^−4^), 1.03 × 10^−3^ (SD, ±5.89 × 10^−4^), 8.66 × 10^−4^ (SD, ±5.65 × 10^−4^), and 1.68 × 10^−3^ (SD, ±1.01 × 10^−3^) in the HC, PDND, PD-MCI, and PDD groups, respectively (Figure 2). PDD showed a statistically significantly increased ratio of miR-203a-3p/miR-16-5p compared to PD-MCI (*p*-value = 0.0006) and PDND (*p*-value = 0.0407). However, no significant difference was observed in the ratio of miRNA when comparing PDND with PD-MCI. This finding may result from the minimal cognitive decline from PDND to PD-MCI, which may be insufficient for detecting the altered expression of plasma miRNA. No significant difference was seen in the ratio of miR-203a-3p/miR-16-5p between HC, PDND, PD-MCI, and PDD individually.

#### 2.2.3. Correlation of miRNA Expression and Cognitive Performance

To determine whether the selected miRNAs were associated with cognitive impairment, the correlations between the ratio of miR-203a-3p/miR-16-5p and the total score and individual domain scores of MoCA were analyzed using Spearman correlation analysis (Table 3, Figure S4). No significant correlation was observed between the ratio of miR-203a-3p/miR-16-5p and gender, age, education level, duration, Hoehn–Yahr stage, UPDRS III, or LEDD (*p*-value > 0.05) in the PD patients. After performing the Spearman correlation analysis, the ratio of miR-203a-3p/miR-16-5p showed a significant negative correlation with the total MoCA score (r = −0.237, *p*-value = 0.024) in the PD patients (Table 3). To determine the specific cognitive domains that were closely related to the miRNA ratio, different cognitive domains of MoCA, such as the visuospatial, naming, attention, language, abstraction, memory, and orientation domains, were analyzed. The results show that the ratio of miR-203a-3p/miR-16-5p had a significant correlation with three MoCA domains, namely the visuospatial, language, and orientation domains (Table 3).

#### 2.2.4. Using the Ratio of miR-203a-3p/miR-16-5p as a Variable for Building the Regression Model

Using the ratio of miR-203a-3p/miR-16-5p alone, an ROC analysis was performed to show the diagnostic power of PD with cognitive decline in PD patients. The results of the corresponding ROC analyses for each comparison group analyzed using logistic regression are summarized in Table S1. The 95% confidence intervals of the sensitivity, specificity, and accuracy of each set of compared groups were estimated, followed by ROC analyses (Table S1). The ROC curve analysis discriminating between PD-MCI and PDD showed an AUC of 0.716 (95% CI, 0.432–0.951, Table S1). In addition, the ROC curve analysis discriminating between PDND and PDD showed an AUC of 0.741 (95% CI, 0.482–0.951, Table S1). Both ROC analyses support the notion that the ratio of miR-203a-3p/miR-16-5p could be used to predict PDD (total MoCA score ≦ 21).

To determine whether the demographic variables may also serve as confounding factors for the differentiation of PDD (total MoCA score ≤ 21) from PwP, multivariate logistic regression models were used. A full regression model was developed, with predictor variables including age, gender, onset age, years spent in formal education, UPDRS III score, and the ratio of miR-203a-3p/miR-16-5p. The total MoCA score was defined as the response variable. The Akaike Information Criterion (AIC) value of each regression model was estimated for the goodness of fit.

The results show that the reduced model with three variables, namely the ratio of miR-203a-3p/miR-16-5p, age, and the UPDRS III score, presented the best performance for predictions of PDD (AIC = 63.20). The results show that the ratio of miR-203a-3p/miR-16-5p, age, and the UPDRS III score all have positive associations with PDD (Table S2). In other words, an older age and more severe motor symptoms with a higher ratio of miR-203a-3p/miR-16-5p could be associated with a higher risk of PDD (Table S2)

**Figure 3:**
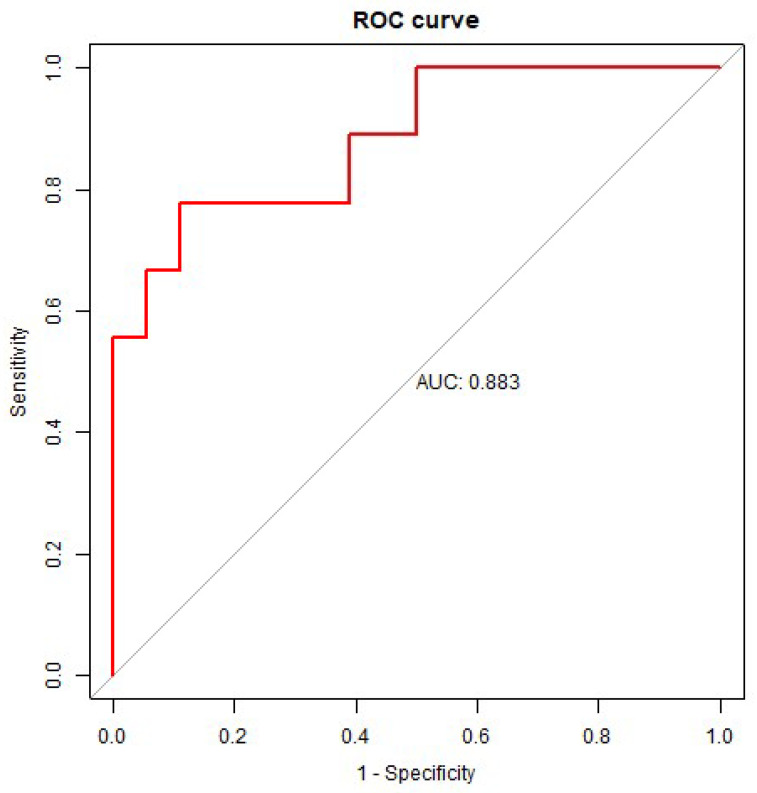
The ROC curve of the logistic regression model with three variables, including the ratio of miR-203a-3p/miR-16-5p, age, and the UPDRS III score. The AUC was estimated with the 30% test dataset. ROC curve, receiver operating characteristic curve; AUC, area under the ROC curve; UPDRS III, the part III of the Unified Parkinson’s Disease Rating Scale.

After variable selection, an ROC analysis of the test dataset was conducted to examine the power of the reduced model with three variables, including the ratio of miR-203a-3p/miR-16-5p, age, and the UPDRS III score (Table 4). The corresponding ROC curve is shown in Figure 3. Apart from the abovementioned model, other ROC analyses were also performed for the regression models without the ratio of miR-203a-3p/miR-16-5p to examine whether the ratio of miR-203a-3p/miR-16-5p serves as a predictive parameter for PDD (Table 4). The ROC analysis and the 95% confidence intervals of the AUC, specificity, sensitivity, and accuracy of the logistic regression models are summarized in Table 4. As a result, the reduced logistic regression model for predicting PDD showed an AUC of 0.8827 (95%CI, 0.7282–0.9938), a sensitivity of 0.7778, a specificity of 0.8889, and an accuracy of 0.8519 (Table 4). The results show that the regression model with three variables, namely the ratio of miR-203a-3p/miR-16-5p, age, and UPDRS III, had a higher prediction performance (AUC = 0.8827) than the regression model with two variables, namely age and UPDRS III (AUC = 0.8272).

#### 2.2.5. MiR-203a-3p Associated with Cognition-Related KEGG Pathways

To elucidate the role of miR-203a-3p in the cognition-related pathological mechanisms of PD, the target genes and the involved molecular pathways were filtered according to experimental evidence (Table S3). Three non-cancer-related pathways and the predicted target genes of miR-203a-3p are summarized in Table 5. As a result, a KEGG analysis of miR-203a-3p revealed several possible pathways that may be associated with the pathology of PD with cognitive dysfunction, including the dopaminergic synapse, apoptosis, and NF-kappa B signaling pathways.

## 3. Discussion

In this study, we first selected six miRNAs using the BOLD selector analysis scheme taken from the NGS profiling dataset in the discovery cohort. Khoo et al. suggested that the expression level of plasma miR-626 was downregulated in PwP compared to HC examined using TaqMan RT-PCR [31], but no significant difference was observed in our discovery cohort between PDND and HC using NGS. The different findings may be results of the different cohorts or tools used for miRNA measurement between RT-PCR and NGS. In the validation phase of the current study, only miR-203a-3p was found to be significantly increased in patients with dementia as compared with the PD-MCI and PDND groups. In a current study, we demonstrated the significant increase in PDD compared to the other PD groups. No statistically significant differences were observed in the miRNA ratios of HC between each PD group. The *p*-value was large in HC vs. PDND (*p*-value > 0.9999), HC vs. PD-MCI (*p*-value = 0.1228), and HC vs. PDD (*p*-value = 0.6731) groups. Additionally, no statistically significant difference was found between PD-MCI and PDND (*p*-value > 0.9999). Thus, the miR-203a-3p/miR-16-5p ratio may not be a suitable marker for diagnosing PD-MCI from PDND. In patients with PD, the miR-203a-3p/miR-16-5p ratio significantly increased in PDD as compared to PD-MCI and PDND. We speculated that PDD patients may be more vulnerable than others to the pathological changes in miR-203a-3p/miR-16-5p concentrations, which could potentially contribute to neurodegeneration. Of note, the ratio of miR-203a-3p/miR-16-5p had a significant negative correlation with the total score and the three MoCA domains, namely the visuospatial, language, and orientation domains. According to previous studies, these three cognitive domains are associated with the frontal lobe’s functions, which correspond to the pathological brain region of patients of PD with cognitive dysfunction [32]. Apart from poor executive function, diminished visuospatial and language functions have also been highlighted in PD-MCI and PDD as features of motor and cognitive decline symptoms [33,34]. Overall, these findings support our hypothesis that miR-203a-3p may serve as a biomarker of global and domain-specific cognitive decline in PwP. To understand the specificity of miR-203a-3p in detecting cognitive decline in AD, the most common cause of dementia in elderly people, we preliminarily enrolled 15 cases diagnosed with AD [35] and measured their plasma miR-203a-3p via ddPCR. The preliminary results showed no significant difference in the ratio of miR-203a-3p/miR-16-5p between AD and PDD (Figure S5). Furthermore, no significant correlation was observed between the MoCA score and the ratio of miR-203a-3p/miR-16-5p in the group of AD patients.

Various studies have shown an association between miRNAs and neurodegenerative diseases, including Alzheimer’s disease (AD), frontotemporal dementia, and PD [36,37,38,39]. For instance, Khoo et al. found four upregulated miRNAs, including miR-1826/miR-450b-3p (paired), miR-626, and miR-505, and used them to differentiate PwP from normal controls. This process used a combination of a microarray for discovery and TaqMan RT-PCR in the replication dataset to assess the expression of miRNAs [31]. The results showed a highest predictive performance with a sensitivity of 0.91 and a specificity of 1 [31]. In brief, some other miRNAs can be used as biomarkers to distinguish PD from HC. For instance, Ravanidis et al. measured the human-brain-enriched miRNA expression level via RT-PCR in a discovery cohort of 100 HC, 99 idiopathic PD, and 53 PD patients carrying genetic mutations [29]. The author further validated the differentially expressed miRNAs using RT-PCR in the plasma of 92 HC and 109 idiopathic PD patients [40]. The results showed that miR-22-3p, miR-124-3p, miR-136-3p, miR-154-5p, and miR-323a-3p were significantly upregulated in PD compared to HC after pooling the dataset of the discovery and validation studies together [40]. In assessing the combination of the 3 miRNAs, including miR-7-5p, miR-136-3p, and miR-409-3p, age and gender together obtained an AUC of 0.736, a sensitivity of 0.72, and a specificity of 0.67 for the merged dataset [40]. Other potential miRNA biomarkers for identifying PD from HC were summarized in our previous review article [17]. MiRNAs are also promising biomarkers of PD with cognitive decline due to their critical roles in synaptic plasticity, memory, and the regulation of neurodegenerative disease-associated proteins such as α-syn, amyloid-β, and tau [41,42]. Han et al. found a significantly decreased level of miR-29s in patients with 22 PDD compared to those in 39 PD with normal cognition (AUC, 0.859; 95% CI, 0.782–0.937) when measuring using RT-PCR [43]. In the current study, which discriminated PDD from non-PDD (PD-MCI and PDND) using the ratio of miR-203a-3p/miR-16-5p alone (Table 4), the AUC was 0.759 (95%CI, 0.537–0.938). The differential power (AUC, 0.883; 95%CI, 0.728–0.994) for distinguishing PDD patients from other PD patients, determined using the miRNA ratio, age, and the UPDRS III score together (Table 4), performed better than that of Han et al. [43]. MiR-203a-3p belongs to the miR-203 miRNA family. It has been shown that miR-203a-3p binds to the 3′UTR of *SNCA*, encodes the α-syn protein, leads to the overexpression of a-syn, and increases the aggregation and spreading of the a-syn protein, effects which have been reported to be related to the pathogenesis of PD with dementia [44,45]. Moreover, miR-203a-3p has been suggested to bind to the 3′UTR of human DJ-1 mRNA, which is a Parkinson’s disease-related protein. DJ-1 may protect neurons from cytotoxic oxidative damage in its physiological function [46,47,48]. The overexpression of miR-203a-3p has been suggested to cause a deficiency of DJ-1 and further result in oxidative-stress-induced cell death [46,47], microglia-regulated neuronal injury [49], and the promotion of the neurodegenerative phenotype in vivo [50]. The above findings suggest that miR-203a-3p might contribute to the underlying mechanism related to cognitive impairment in PD and support the hypothesis of selecting miR-203a-3p as a biomarker for the diagnosis of PDD.

In addition to the aforementioned studies, it has been proposed that the underlying genes regulated by miR-203a-3p are related to cognitive decline in PwP. The KEGG analysis showed that multiple target genes of miR-203a-3p consisted of pathways, including the dopaminergic synapse, apoptosis, and NF-kappa B signaling pathways (Table 5). The loss of dopaminergic synapses in the substantia nigra is assumed to be a hallmark of the progression of motor symptoms in PwP [2]. Severe motor dysfunctions in PD patients have been observed to be associated with PDD. Our data also demonstrated that patients with PDD have a higher UPDRS III motor score compared to patients without cognitive impairment. The evidence supports the existence of a relationship between increased miR-203a-3p and worse dopaminergic neuronal losses. It is noteworthy that PDD is not only characterized by the aggregation of fibril α-syn, but also by tau and amyloid plaque pathologies [6]. Swarup et al. detected the activation of the apoptotic pathway via caspase-8 protein expression, resulting from the overexpression of miR-203, in both primary cortical mouse neuronal cultures and Tg4510 tau transgenic mice [50]. In addition, evidence has suggested that miR-203 is upregulated in the human frontal cortex of patients with frontal temporal dementia (FTD) compared to control samples [48]. Overexpressed miR-203 may also result in neuroinflammation and neuronal cell death in the hippocampi of mice, which were associated with spatial learning and memory dysfunction in the Barnes maze test by Li et al. [51]. The authors suggested that the overexpressed miR-203 in both BV2 cells and the mouse hippocampus resulted in reduced protein expression of 14-3-3θ, which is the inhibitor of TLR2-induced NF-κB signaling [51], which may led to the activation of neuroinflammation and neuronal cell death.

There were some limitations to this study. The sample size was limited, and so a larger sample size and a follow-up study are needed. Furthermore, since PDD was associated with a higher age in our study (Table 2 and Table 4), aging’s effect on the level of miR-203a-3p was uncertain. Finally, this study only measured the plasma cell-free miRNAs, including different fractions of miRNAs, such as the extracellular-vesicle-encapsulated miRNAs and the non-vesicle-associated miRNAs [19]. Exosome-derived miRNA extraction normally requires more preparation than plasma miRNA extraction due to the low yields and the extra separation and purification steps required [52]. Hence, cell-free plasma miRNAs are preferred when the size of the sample is limited.

## 4. Materials and Methods

### 4.1. Plasma miRNA Profiling in the Discovery Cohort

#### 4.1.1. Recruitment of Participants

All patients with PD met the inclusion criteria proposed in the UK Parkinson’s Disease Society Brain Bank Criteria. Participants receiving treatments or who had a history of the following criteria were excluded from this study: (1) cancer; (2) server cardiovascular disease, renal disease, or brain injury; (3) autoimmune disease; (4) psychiatric disorders, such as schizophrenia; (5) deep brain stimuli surgery; (6) known genetic mutations associated with Parkinson’s disease; (7) difficulty in blood clotting; and (8) secondary and atypical Parkinsonism or other possible cognition-affected neurological and musculoskeletal disorders, including multiple-system atrophy, progressive supranuclear palsy, corticobasal degeneration, and dementia with Lewy bodies. The cognitive function of PD-MCI and PDD was evaluated using the total score of the Mini-Mental Status Examination (MMSE) [53,54], and these were found to be from 25 to 26 and below 25, respectively. A total of 174 participants, comprising 40 HCs, 51 patients with MSA, 37 with PDND, 23 with PD-MCI, and 23 with PDD, were recruited. The current study focused on the findings for patients with PD; the analysis of patients with MSA will be discussed in another publication. The participants were enrolled at the Centre for Parkinson and Movement Disorders, National Taiwan University Hospital, a referred tertiary medical center in Taipei.

#### 4.1.2. Plasma Collection

Ten milliliters of blood was collected in a BD Vacutainer^®^ K2E (EDTA) Plus Blood Collection Tubes (Becton, Dickinson and Company, Franklin Lakes, NJ, USA) device and centrifuged at 2200× *g* for 15 min at room temperature (swinging bucket, KUBOTA 4000, Tokyo, Japan) within 3 h of collection. The plasma layer was transferred, mixed via pipetting, and stored at −80 °C until follow-up experiments were performed.

#### 4.1.3. Plasma miRNA Sequencing

Samples containing small RNAs (<200 nucleotides) derived from 200–400 μL of human plasma were prepared using the Qiagen miRNeasy Mini kit (Qiagen, Hilden, Germany; #217004). A small RNA-seq library was constructed using the QIAseq miRNA Library Kit, which incorporated unique molecular identifiers (UMIs) (Qiagen, #331502). Single-ended small RNA-seq with a read length of 75 bp was performed on Illumina MiSeq from DNA Chip Research Inc. (Tokyo, Japan), followed by adaptor trimming on a Qiagen Cloud System with FastQC quality control and UMI deduplication. We based the definition of the small RNA sequences as miRNA species and their annotations on their alignment with the miRNA reference database miRBase (release 21). To normalize the miRNA raw read counts across samples, a scaling method was implemented based on a weighted trimmed mean of the log_2_ expression ratios (trimmed mean of M values, TMM) [55]. In order to merge the two miRNA datasets (containing 75 and 99 samples; the datasets excluding patients with MSA are presented in Tables S4 and S5, respectively), a surrogate variable analysis in R (SVA; V.3.48) was used to normalize and remove batch effects. The full processed dataset, excluding diagnostic subjects that were not analyzed in this study, is presented in Table S6.

#### 4.1.4. BOLD Selector including a Data Analytics Scheme

In the miRNA data analysis, we carried out a three-stage process. The initial stage involved data preprocessing. Missing values were approximated as 0. In order to merge the two datasets (containing 75 and 99 reads), a surrogate variable analysis in R (SVA; V.3.48) was used to normalize and remove batch effects. The union of the lowest expressed 10% of microRNAs in both batches was trimmed before the statistical analysis. The BOLD selector included a data analytics scheme [21,56], narrowing down the ranking of miRNAs with an adjusted parameter δ, which was suitable for clustering biomarker selection in supersaturated data. The second stage focused on the cross-validation of the dataset using the BOLD selector. Prior to this stage, we standardized the expression matrix X and centered response variable Y, which were originally coded as 0 and 1. We chose the best δ from 15 uniformly spaced cut-points within an interval from 0 to the maximum absolute value of max|X^T^ Y|. Data were split into 5 parts for cross-validation, with 80% being used for training and the remaining 20% being used for testing in each part. We applied the BOLD selector to the training data and constructed a logistic regression formula based on the selected microRNA candidates in order to predict the testing data. The model’s fit across 5 testing folds was assessed using the average area under the receiver operating characteristic curve (AUC), enabling us to select the optimal tuning parameter with the highest average AUC. The final stage entailed performing a full data analysis on the tuning parameters that fell between the best δ and max|X^T^ Y|. During this phase, we ranked and identified the most important factors, which were subsequently employed to construct a final logistic regression formula.

#### 4.1.5. Statistical Analysis

The demographic variables in the discovery phase and the NGS read counts of the 6 miRNAs were analyzed using GraphPad Prism (version 7.04). The demographic variables analyzed using non-parametric one-way ANOVA, namely Kruskal–Wallis tests, included gender and age. After performing the Kruskal–Wallis tests, Dunn’s multiple comparisons tests were conducted for a post hoc analysis in order to reveal whether there were significant differences between the study groups. The heat map plot, representing the NGS reads of the 6 miRNA candidates in the 4 study groups, was visualized via Python (version 3.10.12), Matplotlib (version 3.7.1) [57], and Seaborn libraries (version 0.12.2) [58]. The fold change in miRNA reads was calculated by log_2_× (the mean of the patient group with cognitive decline/the mean of the patient group without cognitive decline).

### 4.2. Validating Plasma miRNA Candidates in New PD Cohort

#### 4.2.1. Sample Size Estimation

Two miRNAs were measured in 4 study groups: a group with Parkinson’s disease with no dementia (PDND); one with Parkinson’s disease with mild cognitive impairment (PD-MCI); one with Parkinson’s disease with dementia (PDD); and a healthy control (HC, as a control group) group. We performed prior sample size estimation using G-power 3.1.9.4 [59], using the statistic F test of ANOVA (fixed effects, special effects, main effects, and interactions), effect size = 0.5, power = 0.8, and α = 0.05. The total sample size was suggested to be 48 or above. Considering the greater statistical power and in order to avoid heterogeneity within each study group, a total of 120 participants were recruited and examined.

#### 4.2.2. Recruitment of Participants

All PD patients met the same inclusion and exclusion criteria used in recruitment for the discovery phase. In addition, the Unified Parkinson’s Disease Rating Scale (UPDRS) part III was used to evaluate the motor functions of PDND, PD-MCI, and PDD. PD-MCI and PDD achieved the criteria of the Montreal Cognitive Assessment (MoCA), a level I cognitive assessment proposed by the MDS Task Force [4,10]. The levodopa equivalent daily dose (LEDD) was calculated for PD patients via the LEDD conversion factor [60]. Moreover, demographic variables, including gender, age, the duration of the disease, UPDRS III score, total MoCA score, years spent in formal education, and LEDD, were collected for each participant. The daily dose of anticholinergic drugs, including Akinfree and Biperiden, was estimated for each PD patient if used.

Eventually, 30 patients with PDND, 34 patients with PD-MCI, and 30 patients with PDD were recruited from the Centre for Parkinson and Movement Disorders at the National Taiwan University Hospital. Moreover, 30 HCs were recruited from the National Taiwan University Hospital and the Shixiang Community in Taiwan. All subjects signed informed consent forms for inclusion before they participated in the study.

#### 4.2.3. Cognitive Assessments

All participants underwent the Montreal Cognitive Assessment (MoCA) [4] to quickly determine their cognitive performance, and this was carried out by Y.F. Hsu. The cognitive domains, including visuospatial, naming, attention, language, abstraction, memory, and orientation domains were evaluated via MoCA, and the total score was used for grouping. HC and PDND should have a total MoCA score equal to or above 26. PD-MCI should have a total MoCA score ranging from 22 to 25. PDD should have a total MoCA score equal to or below 21.

#### 4.2.4. Plasma Collection

Plasma collection followed the protocol mentioned for the discovery cohort.

#### 4.2.5. RNA Extraction

Small RNAs were extracted from 200 μL of plasma using the miRNeasy Serum/Plasma Advanced kit (Qiagen, Hilden, Germany). The extraction process generally followed the manufacturer’s instructions, with several modifications listed below. In order to test whether the extraction efficiency was robust, the exogenous synthetic spike-in UniSp6 (Qiagen, Hilden, Germany) was added to the lysis buffer (Figure S1). However, UniSp6 is recommended for measuring extraction efficiency and is not suitable for the normalization of miRNA expression levels according to the manufacturer’s instructions. Thawed plasma samples underwent a series of centrifugation procedures: first, they were centrifuged at 12,000× *g* at 4 °C for 3 min (fixed-angled), and then they were further centrifuged at 12,000× *g* (fixed-angled) at room temperature for 30 s, 30 s, 30 s, 2 min, and 5 min. Twenty-two microliters of 55 °C pre-warmed RNase-free water (Invitrogen, Life Technologies Corporation, Grand Island, NY, USA) was added for RNA elution. Additionally, the eluted RNA was transferred to and incubated in a UCP MiniElute column (Qiagen, Hilden, Germany) for 10 min at room temperature. After centrifugation at 12,000× *g* for 1 min (fixed-angled, KUBOTA 3300T, Tokyo, Japan), the final RNA was immediately placed on ice for reverse transcription (RT). The cDNA synthesis was performed following the instructions of the miRCURY LNA miRNA SYBR Green kit (Qiagen, Hilden, Germany). The mixture prepared for cDNA synthesis contained 10 μL of extracted RNA, 2 μL of 10× miRCURY RT Enzyme Mix, 4 μL of 5× miRCURY SYBR Green RT Reaction Buffer, and 4 μL of RNase-free water (Invitrogen, Thermo Fisher). After the thermal reaction cycle was complete, the cDNA sample was stored at −20 °C until ddPCR examination.

#### 4.2.6. Droplet Digital PCR

For the examination of miR-203a-3p and miR-16-5p, the cDNA samples were diluted to 1:10 and 1:320, respectively. One non-RT control (no transcriptase added during RT) and one non-template control (no cDNA template added during ddPCR amplification; NTC) were quantified to ensure that no genomic DNA remained and that no false-positive results would be detected. miRNA was quantified using a ddPCR system (Bio-Rad Laboratories, Inc., Hercules, CA, USA). First, a mixture with a total volume of 20 μL was prepared with 9 μL of diluted cDNA, 10 μL of digital PCR™ Eva-Green supermix (Bio-Rad, Hercules, CA, USA), and 1 μL of LNA miRCURY miRNA PCR Assay (for miR-203a-3p and miR-16-5p) (Qiagen, Hilden, Germany). Second, 70 μL of QX100 Droplet Generation oil (Bio-Rad, Hercules, CA, USA) and 20 μL of the mixture were loaded into a cartridge. After they being processed in the QX200 Droplet Generator (Bio-Rad, Hercules, CA, USA), the droplet-containing liquid was gently transferred onto a 96-well plate, and cDNA amplification was initiated in a T100 thermal cycler (Bio-Rad, Hercules, CA, USA). The thermal cycling was modified from the manufacturer’s instructions (Bio-Rad, Hercules, CA, USA). The annealing temperature (Tm) was adjusted individually for miR-203a-3p (Tm = 55 °C) and miR-16-5p (Tm = 56 °C). The PCR products were quantified using the QX200 Droplet Reader (Bio-Rad, Hercules, CA, USA) and QuantaSoft software (version 1.7, Bio-Rad, Hercules, CA, USA). The quality control of the ddPCR result was suggested to meet the criteria of a total droplet number above 10,000 copies/μL and a total number of positive droplets above 3 copies/μL. The copy number per microliter of miR-203a-3p was divided by that of miR-16-5p to obtain the ratio of miRNA.

#### 4.2.7. Pathway Prediction

Designed based on a well-known database that integrates up-to-date genomic information, Kyoto Encyclopedia of Genes and Genomes (KEGG) pathway analysis provides graphic maps for genes of interest, enabling the interpretation and prediction of molecule cascades and potential functions [61,62,63]. Hence, the target genes of miR-203a-3p were predicted using the miRPathDB 2.0 [64], and the predicted pathways were filtered using the experimental (any) data resources.

#### 4.2.8. Statistical Analysis

The demographic variables and the ratio of miR-203a-3p/miR-16-5p were analyzed using GraphPad Prism (version 7.04). Outlier detection for the ratio of miR-203a-3p/miR-16-5p in each study group was conducted by means of the IQR-based method, where IQR represents the difference between the first (Q1) and the third quartiles (Q3) [65]. The data in each group were sorted by ascending order, and the data points which were larger than the third quartile by 1.5 times the IQR (>Q3 + 1.5 × IQR) or smaller than the first quartile by 1.5 times the IQR (<Q1 − 1.5 × IQR) were identified as outliers. Thus, 4 outliers were removed in PD-MCI and the remaining clean dataset was used for the statistical analysis. The demographic variables analyzed using the non-parametric one-way ANOVA Kruskal–Wallis tests included gender, age, total MoCA score, duration of disease, UPDRS III score, years spent in formal education, and LEDD. After performing the Kruskal–Wallis tests, Dunn’s multiple comparisons tests were conducted for a post hoc analysis to reveal whether there were significant differences between the study groups. Spearman correlation tests, a form of non-parametric correlation analysis, were applied for the ratio of miR-203a-3p/miR-16-5p, different cognitive domains (visuospatial, naming, attention, language, abstraction, memory, and orientation domains), and the demographic variables. In order to understand the diagnostic power of analyzing PD with or without cognitive decline using the ratio of miR-203a-3p/miR-16-5p, the 4 study groups were compared and analyzed via receiver operating characteristic (ROC) curve analysis with R software (version 4.3.2), package pROC (version 1.18.5) [66], and package caret (version 6.0–94) [67]. In addition, multivariate logistic regression models were developed that consisted of the demographic variables and the ratio of miRNA factors. The demographic and miRNA data of 90 PD patients were divided into two groups, with 70% used for model training and 5-fold cross-validation and the remaining 30% used for model testing. A reduced logistic regression model was determined via the smallest Akaike Information Criteria (AIC). The predicted values of the AUC were estimated using the 30% test dataset. All coordinates of ROC curves, including the area under the curve (AUC), sensitivity, specificity, and accuracy, were estimated via the maximal sum (sensitivity + specificity). Additionally, 95% confidence intervals (CIs) were also calculated for the AUC, sensitivity, specificity, and accuracy using bootstrapping (boot runs = 2000).

## 5. Conclusions

In summary, plasma miR-203a-3p was significantly increased in the PDD group compared to the PD-MCI or PDND groups. Moreover, the ratio of miR-203a-3p/miR-16-5p was negatively correlated with the total MoCA score and multiple domains, such as the visuospatial, language, and orientation domains. The logistic regression model (AUC = 0.883), combining age, the ratio of miRNA, and UPDRS III, may facilitate the differentiation of PDD from PwP. MiR-203a-3p, as a plasma-based biomarker used for predicting the diagnosis of PDD, needs to be verified further in larger clinical studies.

## Figures and Tables

**Table 1:** Demographic characteristics of each study group in the discovery cohort.

	HC (*n* = 40)	PDND (*n* = 37)	PD-MCI (*n* = 23)	PDD (*n* = 23)	*p*-Value *
Gender, % male	40.00%	54.05%	73.91%	52.17%	ns
Age, year	69.08 ± 6.05	64.78 ± 12.51	67.70 ± 7.15	72.00 ± 5.52	ns

* *p*-value was estimated by performing Kruskal–Wallis tests on the 4 groups. Data are shown as the mean ± standard deviation (SD). HC, healthy control; PDND, Parkinson’s disease with no dementia; PD-MCI, Parkinson’s disease with mild cognitive impairment; PDD, Parkinson’s disease with dementia; ns, no significant difference.

**Table 2:** Demographic characteristics of each study group in the validation cohort.

	HC (*n* = 30)	PDND (*n* = 30)	PD-MCI (*n* = 30)	PDD (*n* = 30)	*p*-Value *
Gender, % male	56.67%	56.67%	53.33%	46.67%	-
Age, year	66.67 ± 5.14	69.67 ± 7.03	70.13 ± 6.75	75.20 ± 6.92	<0.0001
MoCA ^†^	28.00 ± 2.00	28.00 ± 1.25	23.00 ± 1.00	17.50 ± 7.00	<0.0001
Education, year	14.13 ± 4.13	14.13 ± 2.79	11.47 ± 4.75	10.73 ± 4.64	0.0049
Onset age, year	-	63.53 ± 7.96	64.13 ± 7.96	67.37 ± 8.71	ns
Duration, year	-	7.10 ± 3.91	6.90 ± 3.07	7.23 ± 4.75	ns
Hoehn–Yahr stage ^†^	-	2.00 ± 1.00	2.00 ± 1.00	3.00 ± 2.00	<0.0001
UPDRS III ^†^	-	13.00 ± 12.00	18.50 ± 9.00	27.00 ± 22.00	<0.0001
LEDD	-	682.54 ± 438.75	747.78 ± 398.03	765.82 ± 419.36	ns

HC, healthy control; PDND, Parkinson’s disease with no dementia; PD-MCI, Parkinson’s disease with mild cognitive impairment; PDD, Parkinson’s disease with dementia. * *p*-value was estimated by performing Kruskal–Wallis tests on the 4 groups. ns: no significant difference. Data for gender, age, education, onset age, duration and LEDD are shown as the mean ± SD. LEDD, levodopa equivalent daily dose. ^†^ Data for MoCA, Hoehn–Yahr stage, and UPDRS III are shown as the median ± IQR. MoCA, Montreal Cognitive Assessment; UPDRS III, part III of the Unified Parkinson’s Disease Rating Scale.

**Table 3:** Spearman correlation of the miRNA ratio and cognitive domain in PD patients.

Cognitive Domains of MoCA	Spearman r	*p*-Value
Total score *	−0.237	0.024
Visuospatial *	−0.207	0.050
Naming	−0.117	0.272
Attention	−0.112	0.292
Language *	−0.208	0.049
Abstract	−0.124	0.246
Memory	−0.205	0.052
Orientation *	−0.220	0.037

* The ratio of miR-203a-3p/miR-16-5p had a significantly negative correlation (*p*-value < 0.05).

**Table 4:** The 95% confidence interval of the AUC, specificity, sensitivity, and accuracy for the logistic regression model performed for all of the PD patient groups.

Variables	AUC (95% CI)	Specificity (95% CI)	Sensitivity (95% CI)	Accuracy (95% CI)
miR-203a-3p/miR-16-5p	0.7593 (0.5370–0.9383)	0.7222 (0.5000–0.9444)	0.7777 (0.5528–1.0000)	0.7407 (0.5917–0.8889)
Age	0.7654 (0.5617–0.929)	0.6667 (0.4444–0.8889)	0.7778 (0.4444–1.0000)	0.7037 (0.5185–0.8889)
UPDRS III	0.7932 (0.5709–0.9691)	0.7778 (0.5556–0.9444)	0.8889 (0.6667–1.0000)	0.8148 (0.6667–0.9630)
miR-203a-3p/miR-16-5p + Age	0.8642 (0.6914–0.9877)	0.8333 (0.6667–1.0000)	0.7778 (0.5528–1.0000)	0.8148 (0.6667–0.9630)
miR-203a-3p/miR-16-5p + UPDRS III	0.8827 (0.7344–0.9815)	0.8333 (0.6667–1.0000)	0.7778 (0.4444–1.0000)	0.8148 (0.6667–0.9630)
Age + UPDRS III	0.8272 (0.6049–0.9815)	0.7222 (0.5000–0.8889)	0.8889 (0.6667–1.0000)	0.7778 (0.6296–0.9259)
miR-203a-3p/miR-16-5p + Age + UPDRS III	0.8827 (0.7282–0.9938)	0.8889 (0.7222–1.0000)	0.7778 (0.4444–1.0000)	0.8519 (0.7037–0.9630)

Multivariate logistic regression models were developed that consisted of variables including the ratio of miR-203a-3p/miR16-5p, age, and part III of the Unified Parkinson’s Disease Rating Scale (UPDRS III). The analyzed dataset, obtained from 90 PD patients, was divided into two groups, with 70% used for model training and 5-fold cross-validation and the remaining 30% of the dataset used for model testing. UPDRS III, part III of the Unified Parkinson’s Disease Rating Scale.

**Table 5:** The KEGG pathway analysis and the predicted target genes of miR-203a-3p.

Database	Pathway	*p*-Value	Targets
KEGG	Dopaminergic synapse	3 × 10^−4^	AKT2, CLOCK, CREB1, GNAS, GSK3B, KIF5B, MAPK8, MAPK9, PPP1CB, PRKACB, PRKCA
KEGG	Apoptosis	0.011	AKT2, ATM, MYD88, PIK3CA, PRKACB, TNF
KEGG	NF-kappa B signaling pathway	0.041	ATM, CXCL8, MYD88, SYK, TNF

## Data Availability

The data presented in this study are available upon request from the corresponding author. The data are not publicly available due to the privacy of the participants.

## Supplementary Materials

The following supporting information can be downloaded at https://www.mdpi.com/article/10.3390/ijms25063554/s1.
