# Supplementary material for: Plasma miR-203a-3p as a Novel Predictor of Dementia in Patients with Parkinson’s Disease
Source: Int J Mol Sci. 2024 Mar 21;25(6):3554. doi: 10.3390/ijms25063554 (PMC10970771; doi:10.3390/ijms25063554)
Supplement: Supplementary file 1 [file ijms-25-03554-s001.zip › supplementary_revsied-v2.pdf]

Supplementary Materials

Figure S1.

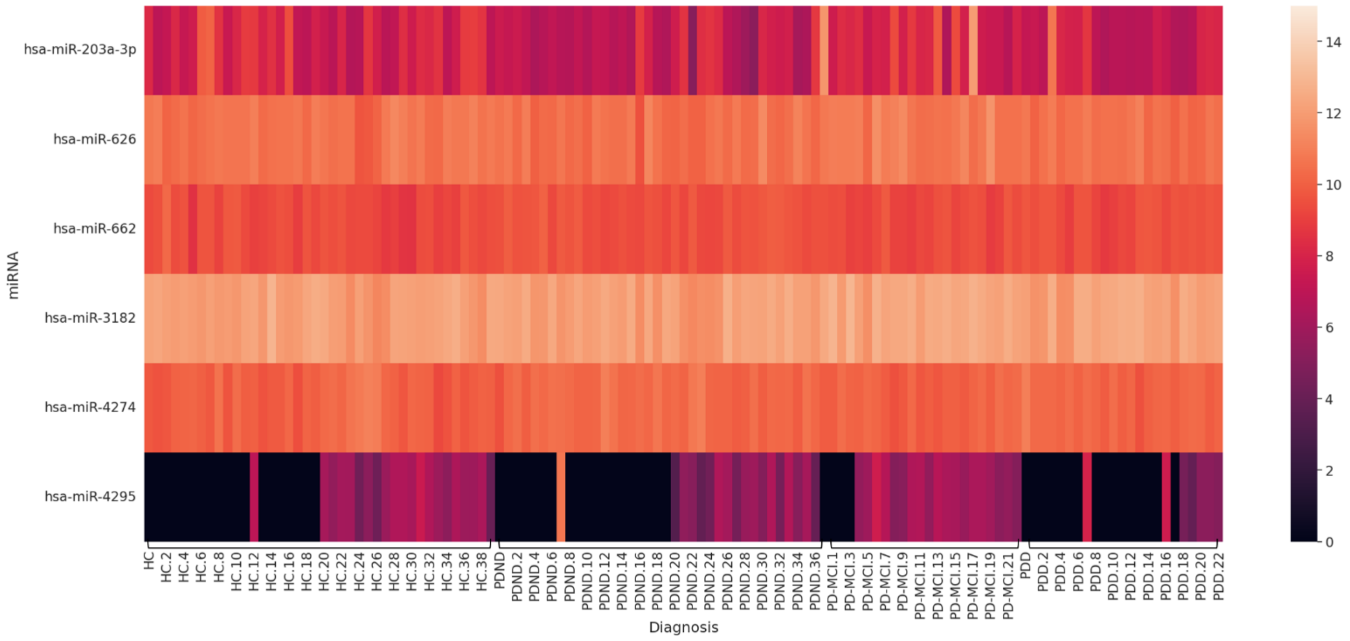

Supplementary Figure 1. The heat map plot of the 6 miRNA included all samples from the 4 study groups (HC, PDND, PD-MCI and PDD) in the discovery phase. The data was represented as Log2(reads). The y-axis indicated the 6 miRNA names; the x-axis indicated the diagnosis group of each sample. HC, healthy control; PDND, Parkinson’s disease with no dementia; PD-MCI, Parkinson’s disease with mild cognitive impairment; PDD, Parkinson’s disease with dementia.

Figure S2.

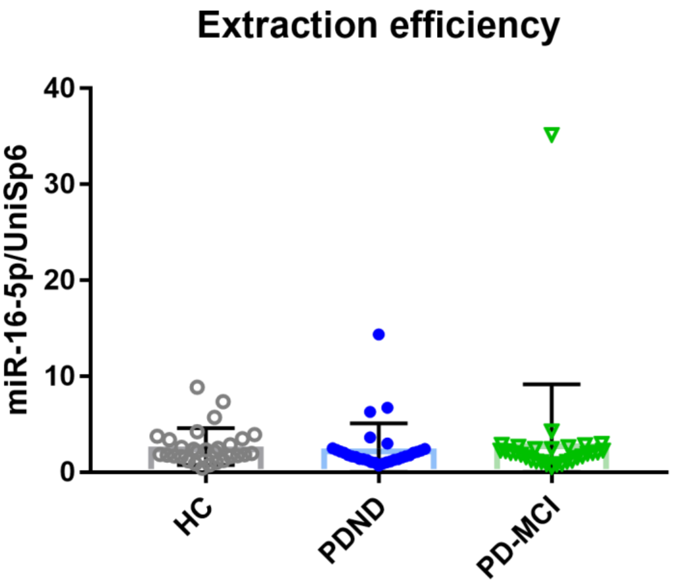

Supplementary Figure 2. The RNA extraction efficiency examination by the ratio of miR-16-5p/UniSp6. The external spike-in UniSp6 (0.5 µl) was added to the lysis buffer before RNA extraction. The expression of miR-16-5p and UniSp6 were examined by ddPCR. The ratio of miR-16-5p/UniSp6 was analyzed using Kruskal-Wallis test followed by Dunn's multiple comparisons test (post-hoc test) among the groups of HC (n=29), PDND (n=30) and PD-MCI (n=30). Data are shown as Mean ± SD. HC, healthy control; PDND, Parkinson's disease with no dementia; PD-MCI, Parkinson's disease with mild cognitive impairment; PDD, Parkinson's disease with dementia.

Figure S3.

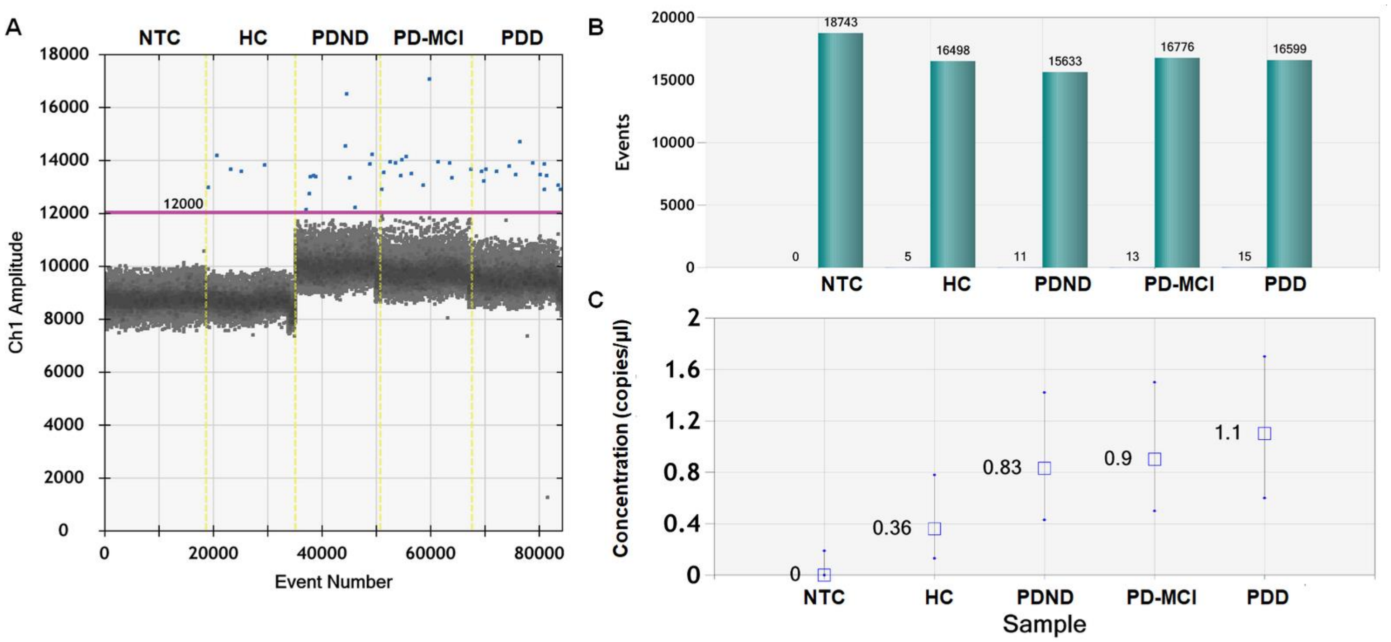

Supplementary Figure 3. The ddPCR examination of miR-203a-3p. **A.–C.** Examples of ddPCR results (n=1 in each study group). **A.** The representative 1D amplitude plot shows the positive (blue) and negative (gray) droplets for examining miR-203a-3p. NTC, the non-template control (n=1), HC (n=1), PDND (n=1), PD-MCI (n=1) and PDD (n=1). The droplets above the amplitude threshold (pink horizontal line) were estimated as positive droplets, and the remaining detected droplets were classified as negative droplets. **B.** The estimated number of total (green bar; 18743, 16498, 15633, 16776 and 16599) and positive (blue bar; 0, 5, 11, 13 and 15) droplets for the examination of miR-203a-3p via ddPCR. Positive droplets with less than 3 droplets were considered negative results. NTC, the non-template control (n=1), HC (n=1), PDND (n=1), PD-MCI (n=1) and PDD (n=1). **C.** The expression level of miR-203a-3p converted to copies per microliter. NTC, the non-template control (n=1), HC (n=1), PDND (n=1), PD-MCI (n=1) and PDD (n=1). HC, healthy control; PDND, Parkinson's disease with no dementia; PD-MCI, Parkinson's disease with mild cognitive impairment; PDD, Parkinson's disease with dementia.

Figure S4.

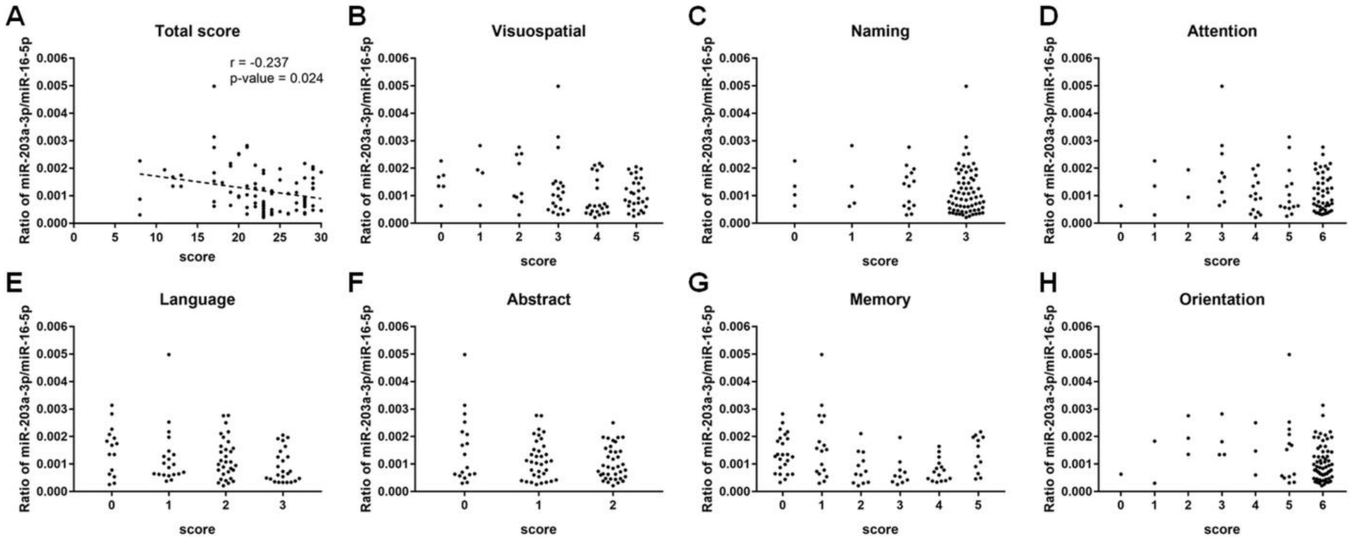

Supplementary Figure 4. The scatter plot of the ratio of miR-203a-3p/miR-16-5p versus cognitive domains used in correlation analysis was conducted by GraphPad Prism (version 7.04). **A-H.** The ratio of miR-203a-3p/miR-16-5p versus cognitive scores, including the total score and individual domain scores of MoCA were analyzed using a Spearman correlation analysis in all PD patients (n=90). **A.** The ratio of miR-203a-3p/miR-16-5p versus the total score of MoCA. **B.** The ratio of miR-203a-3p/miR-16-5p versus the score of visuospatial. **C.** The ratio of miR-203a-3p/miR-16-5p versus the score of naming. **D.** The ratio of miR-203a-3p/miR-16-5p versus the score of attention. **E.** The ratio of miR-203a-3p/miR-16-5p versus the score of language. **F.** The ratio of miR-203a-3p/miR-16-5p versus the score of abstract. **G.** The ratio of miR-203a-3p/miR-16-5p versus the score of memory. **H.** The ratio of miR-203a-3p/miR-16-5p versus the score of orientation.

Figure S5.

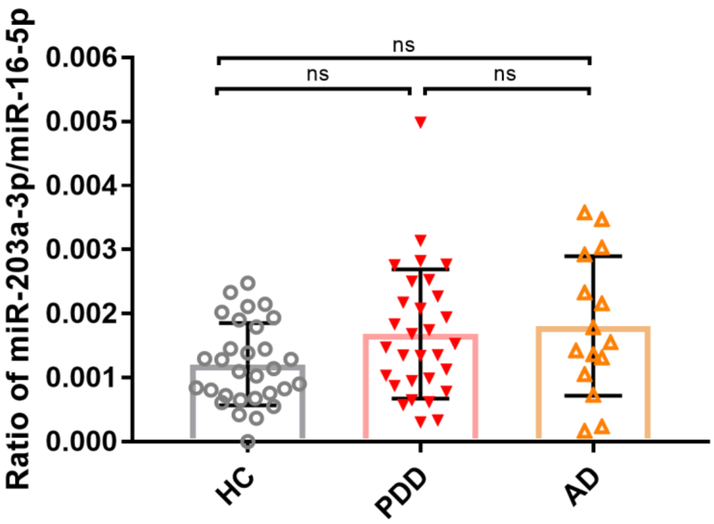

Supplementary Figure 5. The ddPCR examination of miR-203a-3p/miR-16-5p was analyzed by the Kruskal–Wallis test followed by Dunn's multiple comparisons test (post hoc test) among the study groups. ns, no significant difference. HC (n=30), PDD (n=30) and AD (n=15). Data are shown as the mean ± SD. HC, healthy control; PDD, Parkinson's disease with dementia; AD, Alzheimer's disease.

Table S1.

Supplementary Table 1. The AUCs and 95% confidence intervals of specificity, sensitivity and accuracy for the ROC curve analysis for each comparison group using the ratio of miR-203a-3p/miR-16-5p alone.

**Comparison groups**	**AUC (95% CI)**	**Specificity (95% CI)**	**Sensitivity (95% CI)**	**Accuracy (95% CI)**
PD-MCI/PDD	0.7160 (0.4321-0.9506)	0.5556 (0.2222-0.8889)	1.0000 (1.0000-1.0000)	0.7778 (0.6111-0.9444)
PD-MCI/PDND	0.5309 (0.2469-0.8025)	0.8889 (0.6667-1.0000)	0.4444 (0.1111-0.7778)	0.6667 (0.4444-0.8333)
PDD/PDND	0.7407 (0.4815-0.9506)	0.5556 (0.2222-0.8889)	1.0000 (1.0000-1.0000)	0.7778 (0.6111-0.9444)
PDD/HC	0.6420 (0.3333-0.9259)	0.6667 (0.3333-1.0000)	0.7778 (0.4444-1.0000)	0.7222 (0.5000-0.8889)
PD-MCI/HC	0.6667 (0.3824-0.9136)	0.8889 (0.6667-1.0000)	0.5556 (0.2222-0.8889)	0.7222 (0.5556-0.8889)
PDND/HC	0.7160 (0.4318-0.9383)	0.8889 (0.6667-1.0000)	0.6667 (0.3333-1.0000)	0.7778 (0.6111-0.9444)

The analyzed dataset was divided by 70% for model training and 5-fold cross validation, and the remaining 30% for model testing. HC, healthy control; PDND, Parkinson's disease with no dementia; PD-MCI, Parkinson's disease with mild cognitive impairment; PDD, Parkinson's disease with dementia; ROC curve, receiver operating characteristic curve; AUC, area under the ROC curve.

Table S2.

Supplementary Table 2. The multivariate logistic regression model for detecting PDD.

Covariate	Coefficient	Estimated Standard Error	z value	p-value
Intercept	-25.42314	8.42341	-3.018	<0.01
miR-203a-3p/miR-16-5p	0.22671	0.08279	2.738	<0.01
Age	0.23692	0.09185	2.579	<0.01
UPDRS_III	0.19145	0.05594	3.422	<0.001

The multivariate logistic regression model was developed consisting of the variables included the ratio of miR-203a-3p/miR16-5p, age and the part III of the Unified Parkinson's Disease Rating Scale (UPDRS III). The analyzed dataset, obtained from the 90 PD patients, was 70% for model training and 5-fold cross validation, and the remaining 30% for model testing.

Table S3.

Supplementary Table 3. The experimental-based predicted pathways and target genes of miR-203a-3p via KEGG database analysis.

Pathway	p-value	Target genes
Hepatitis B	2.73E-09	*AKT2,BIRC5,CDK6,CREB1,CXCL8,E2F1,E2F3,IL6,JUN,MAPK8,MAPK9,MYD88,PIK3CA,PRKCA,SMAD4,SRC,STAT1,TBK1,TNF*
Pathways in cancer	5.04E-07	*ABL1,AKT2,BIRC5,CDH1,CDK6,CXCL8,E2F1,E2F3,EGLN1,FGF2,FZD2,GSK3B,IGF1R,IL6,JUN,MAPK8,MAPK9,MMP1,NCOA4,PIK3CA,PRKCA,SMAD2,SMAD4,STAT1,VEGFA*
Pancreatic cancer	3.68E-06	*AKT2,CDK6,E2F1,E2F3,MAPK8,MAPK9,PIK3CA,SMAD2,SMAD4,STAT1,VEGFA*
Chagas disease (American trypanosomiasis)	2.75E-05	*AKT2,CALR,CXCL8,GNAS,IL6,JUN,MAPK8,MAPK9,MYD88,PIK3CA,SMAD2,TNF*
Ras signaling pathway	3.09E-05	*ABL1,AKT2,EXOC2,FGF2,IGF1R,MAPK8,MAPK9,PIK3CA,PLD2,PRKACB,PRKCA,RAP1A,RASA2,RASAL2,RGL2,TBK1,VEGFA*
Influenza A	4.16E-05	*AKT2,CXCL8,GSK3B,IL6,JUN,MAPK8,MAPK9,MYD88,PIK3CA,PRKCA,SOCS3,STAT1,TBK1,TNF*
Toll-like receptor signaling pathway	4.16E-05	*AKT2,CXCL8,IL6,JUN,MAPK8,MAPK9,MYD88,PIK3CA,STAT1,TBK1,TNF*
Colorectal cancer	6.55E-05	*AKT2,BIRC5,GSK3B,JUN,MAPK8,MAPK9,PIK3CA,SMAD2,SMAD4*
MicroRNAs in cancer	6.55E-05	*ABL1,ATM,BCL2L2,BMI1,CDK6,E2F1,E2F3,PIK3CA,PRKCA,TP63,TRIM71,VEGFA,ZEB1,ZEB2*
Prolactin signaling pathway	9.75E-05	*AKT2,GSK3B,MAPK8,MAPK9,PIK3CA,SOCS3,SOCS6,SRC,STAT1*
Focal adhesion	1.11E-04	*AKT2,CAV1,GSK3B,IGF1R,JUN,MAPK8,MAPK9,PIK3CA,PPP1CB,PRKCA,RAP1A,RAPGEF1,ROCK2,SRC,VEGFA*
HTLV-I infection	1.23E-04	*AKT2,ATM,CALR,CREB1,E2F1,E2F3,FZD2,GSK3B,IL6,JUN,MAPK8,PIK3CA,PRKACB,RAN,SMAD2,SMAD4,TNF*
Hepatitis C	1.63E-04	*AKT2,CXCL8,GSK3B,IFIT1,MAPK8,MAPK9,PIK3CA,SOCS3,STAT1,TBK1,TNF*
Wnt signaling pathway	2.27E-04	*CXXC4,FZD2,GSK3B,JUN,MAPK8,MAPK9,PRICKLE2,PRKACB,PRKCA,ROCK2,SMAD4,WIF1*
Dopaminergic synapse	3.00E-04	*AKT2,CLOCK,CREB1,GNAS,GSK3B,KIF5B,MAPK8,MAPK9,PPP1CB,PRKACB,PRKCA*
FoxO signaling pathway	3.56E-04	*AKT2,ATM,G6PC,IGF1R,IL6,MAPK8,MAPK9,PIK3CA,SMAD2,SMAD4,SOD2*
ErbB signaling pathway	5.16E-04	*ABL1,AKT2,GSK3B,JUN,MAPK8,MAPK9,PIK3CA,PRKCA,SRC*

Melanoma	5.30E-04	*AKT2,CDH1,CDK6,E2F1,E2F3,FGF2,IGF1R,PIK3CA*
Herpes simplex infection	6.53E-04	*CLOCK,IFIT1,IL6,JUN,MAPK8,MAPK9,MYD88,PPP1CB,SOCS3,STAT1,TBK1,TNF*
Bladder cancer	8.25E-04	*CDH1, CXCL8,E2F1,E2F3,MMP1,VEGFA*
Endocytosis	8.64E-04	*ASAP1,CAV1,CCR5,IGF1R,PARD6B,PIP5K1A,PLD2,RAB22A,RNF41,SH3GLB1,SMAD2,SMURF2,SRC*
Proteoglycans in cancer	8.64E-04	*AKT2,CAV1,FGF2,FZD2,IGF1R,PIK3CA,PPP1CB,PRKACB,PRKCA,ROCK2,SMAD2,SRC,TNF,VEGFA*
Osteoclast differentiation	0.001	*AKT2,CREB1,JUN,MAPK8,MAPK9,PIK3CA,SOCS3,STAT1,SYK,TNF*
TNF signaling pathway	0.001	*AKT2,CREB1,IL6,JUN,MAPK8,MAPK9,PIK3CA,SOCS3,TNF*
Tuberculosis	0.001	*AKT2,CREB1,IL6,MAPK8,MAPK9,MYD88,NFYA,SRC,STAT1,SYK,TNF*
Adherens junction	0.002	*CDH1, IGF1R,SMAD2,SMAD4,SNAI1,SNAI2,SRC*
Fc epsilon RI signaling pathway	0.002	*AKT2, MAPK8,MAPK9,PIK3CA,PRKCA,SYK,TNF*
Glioma	0.002	*AKT2, CDK6,E2F1,E2F3,IGF1R,PIK3CA,PRKCA*
GnRH signaling pathway	0.002	*GNAS,JUN,MAPK8,MAPK9,PLD2,PRKACB,PRKCA,SRC*
Insulin signaling pathway	0.002	*AKT2,G6PC,GSK3B,MAPK8,MAPK9,PIK3CA,PPP1CB,PRKACB,RAPGEF1,SOCS3*
Neurotrophin signaling pathway	0.002	*ABL1,AKT2,GSK3B,JUN,MAPK8,MAPK9,PIK3CA,RAP1A,RAPGEF1*
Non-alcoholic fatty liver disease (NAFLD)	0.002	*AKT2,CXCL8,GSK3B,IL6,JUN,MAPK8,MAPK9,PIK3CA,SOCS3,TNF*
Pertussis	0.002	*CXCL8, IL6,JUN,MAPK8,MAPK9,MYD88,TNF*
Rap1 signaling pathway	0.002	*AKT2,CDH1,FGF2,GNAS,IGF1R,PARD6B,PIK3CA,PRKCA,RAP1A,RAPGEF1,SRC,VEGFA*
Renal cell carcinoma	0.002	*AKT2,EGLN1,JUN,PIK3CA,RAP1A,RAPGEF1,VEGFA*
Chronic myeloid leukemia	0.003	*ABL1, AKT2,CDK6,E2F1,E2F3,PIK3CA,SMAD4*
Non-small cell lung cancer	0.003	*AKT2, CDK6,E2F1,E2F3,PIK3CA,PRKCA*

Amphetamine addiction	0.004	*CREB1, GNAS,JUN,PPP1CB,PRKACB,PRKCA*
HIF-1 signaling pathway	0.004	*AKT2,EGLN1,IGF1R,IL6,LDHA,PIK3CA,PRKCA,VEGFA*
Hippo signaling pathway	0.004	*BIRC5,BMPR1A,CDH1,FZD2,GSK3B,PARD6B,PPP1CB,SMAD2,SMAD4,SNAI2*
Salmonella infection	0.004	*CXCL8, IL6,JUN,MAPK8,MAPK9,MYD88,ROCK2*
Shigellosis	0.004	*ABL1, CXCL8,MAPK8,MAPK9,ROCK2,SRC*
TGF-beta signaling pathway	0.004	*ACVR2B,BMPR1A,SMAD2,SMAD4,SMAD9,SMURF2,TNF*
Fc gamma R-mediated phagocytosis	0.005	*AKT2,ASAP1,PIK3CA,PIP5K1A,PLD2,PRKCA,SYK*
Prostate cancer	0.005	*AKT2, CREB1,E2F1,E2F3,GSK3B,IGF1R,PIK3CA*
Toxoplasmosis	0.005	*AKT2,CCR5,MAPK8,MAPK9,MYD88,PIK3CA,STAT1,TNF*
Adipocytokine signaling pathway	0.006	*AKT2, G6PC,MAPK8,MAPK9,SOCS3,TNF*
Chemokine signaling pathway	0.006	*AKT2,CCR5,CXCL8,GSK3B,PIK3CA,PRKACB,RAP1A,ROCK2,SRC,STAT1*
Type II diabetes mellitus	0.006	*MAPK8, MAPK9,PIK3CA,SOCS3,TNF*
Measles	0.007	*AKT2,CDK6,GSK3B,IL6,MYD88,PIK3CA,STAT1,TBK1*
African trypanosomiasis	0.008	*IL6, MYD88,PRKCA,TNF*
Cell cycle	0.008	*ABL1,ATM,CDK6,E2F1,E2F3,GSK3B,SMAD2,SMAD4*
Estrogen signaling pathway	0.008	*AKT2, CREB1,GNAS,JUN,PIK3CA,PRKACB,SRC*
Jak-STAT signaling pathway	0.008	*AKT2, IL24,IL6,IL7,LIFR,PIK3CA,SOCS3,STAT1*
Amoebiasis	0.009	*CXCL8, GNAS,IL6,PIK3CA,PRKACB,PRKCA,TNF*
Cytokine-cytokine receptor interaction	0.009	*ACVR2B,BMPR1A,CCR5,CXCL8,IL24,IL6,IL7,LIFR,TNF,TNFRSF9,VEGFA*
Pathogenic Escherichia coli infection	0.009	*ABL1, CDH1,NCL,PRKCA,ROCK2*

Epstein-Barr virus infection	0.01	*AKT2,GSK3B,JUN,MAPK8,MAPK9,PIK3CA,PRKACB,RAN,SYK,TBK1*
MAPK signaling pathway	0.01	*AKT2,DUSP5,FGF2,JUN,MAP3K13,MAPK8,MAPK9,PRKACB,PRKCA,RAP1A,RASA2,TNF*
NOD-like receptor signaling pathway	0.01	*CXCL8, IL6,MAPK8,MAPK9,TNF*
Rheumatoid arthritis	0.01	*CXCL8, IL6,JUN,MMP1,TNF,VEGFA*
Apoptosis	0.011	*AKT2, ATM,MYD88,PIK3CA,PRKACB,TNF*
Progesterone-mediated oocyte maturation	0.011	*AKT2, IGF1R,MAPK8,MAPK9,PIK3CA,PRKACB*
Inflammatory bowel disease (IBD)	0.012	*IL6, JUN,SMAD2,STAT1,TNF*
RIG-I-like receptor signaling pathway	0.012	*CXCL8, MAPK8,MAPK9,TBK1,TNF*
VEGF signaling pathway	0.012	*AKT2, PIK3CA,PRKCA,SRC,VEGFA*
mTOR signaling pathway	0.012	*AKT2, PIK3CA,PRKCA,TNF,VEGFA*
Thyroid hormone signaling pathway	0.014	*AKT2,GSK3B,PIK3CA,PRKACB,PRKCA,SRC,STAT1*
Malaria	0.016	*CXCL8, IL6,MYD88,TNF*
Cocaine addiction	0.017	*CREB1, GNAS,JUN,PRKACB*
Epithelial cell signaling in Helicobacter pylori infection	0.017	*CXCL8, JUN,MAPK8,MAPK9,SRC*
PI3K-Akt signaling pathway	0.017	*AKT2,CDK6,CREB1,FGF2,G6PC,GSK3B,IGF1R,IL6,IL7,PIK3CA,PRKCA,SYK,VEGFA*
Melanogenesis	0.022	*CREB1, FZD2,GNAS,GSK3B,PRKACB,PRKCA*
B cell receptor signaling pathway	0.024	*AKT2, GSK3B,JUN,PIK3CA,SYK*
Serotonergic synapse	0.024	*ALOX15,GABRB1,GNAS,HTR2A,PRKACB,PRKCA*
Cholinergic synapse	0.027	*AKT2, CREB1,KCNJ2,PIK3CA,PRKACB,PRKCA*
T cell receptor signaling pathway	0.027	*AKT2, GSK3B,JUN,MAPK9,PIK3CA,TNF*

Adrenergic signaling in cardiomyocytes	0.03	*AKT2,CREB1,GNAS,PIK3CA,PPP1CB,PRKACB,PRKCA*
Vascular smooth muscle contraction	0.03	*EDNRA, GNAS,PPP1CB,PRKACB,PRKCA,ROCK2*
Vibrio cholerae infection	0.031	*GNAS, PRKACB,PRKCA,TJP2*
Viral carcinogenesis	0.031	*CCR5,CDK6,CREB1,JUN,PIK3CA,PRKACB,RASA2,SRC,SYK*
Circadian rhythm	0.033	*CLOCK, CREB1,FBXL3*
Endometrial cancer	0.033	*AKT2, CDH1,GSK3B,PIK3CA*
GABAergic synapse	0.033	*GABRB1, PRKACB,PRKCA,SLC12A5,SRC*
Transcriptional misregulation in cancer	0.037	*ATM, BMI1,CXCL8,IGF1R,IL6,RUNX2,SIX1,ZEB1*
Legionellosis	0.039	*CXCL8, IL6,MYD88,TNF*
Morphine addiction	0.041	*GABRB1, GNAS,PDE7A,PRKACB,PRKCA*
NF-kappa B signaling pathway	0.041	*ATM, CXCL8,MYD88,SYK,TNF*
Prion diseases	0.041	*IL6, PRKACB,PRNP*
Small cell lung cancer	0.041	*AKT2, CDK6,E2F1,E2F3,PIK3CA*
Gap junction	0.042	*GNAS, HTR2A,PRKACB,PRKCA,SRC*
Inositol phosphate metabolism	0.042	*IPMK, PI4K2B,PIK3CA,PIP5K1A*
Tight junction	0.042	*AKT2, CASK,PARD6B,PRKCA,SRC,TJP2*
Antigen processing and presentation	0.046	*CALR, CREB1,NFYA,TNF*
